# Marathon-associated sudden cardiac death: a mini review of risk perception, pathophysiological mechanisms, and prevention strategies

**DOI:** 10.3389/fcvm.2026.1847561

**Published:** 2026-05-28

**Authors:** Xingzhan Zhang, Shaobo Li, Yunjing Mai, Hongzhi Chen, Huanhuan Wu, Chao Li, Ling Zhao, Jie Peng

**Affiliations:** 1 Department of Intensive Care Unit, The People’s Hospital Medical Group of Xiangzhou, Zhuhai, Guangdong, China; 2 Department of Cardiovascular and Metabolism Medicine, The People’s Hospital Medical Group of Xiangzhou, Zhuhai, Guangdong, China

**Keywords:** cardiac arrest, cardiovascular prevention, epidemiology, marathon, sports cardiology, sudden cardiac death

## Abstract

Marathon running is widely promoted for its cardiovascular and overall health benefits, yet rare cases of sudden cardiac death (SCD) during long-distance races continue to raise substantial clinical and public health concerns. Although the absolute incidence of marathon-associated SCD is low, its catastrophic nature necessitates a clearer understanding of risk perception, epidemiological patterns, pathophysiological mechanisms, and preventive strategies. This mini review synthesizes current evidence on the incidence, demographic characteristics, etiological spectrum, and temporal features of cardiac arrest and SCD during marathon running. Available data indicate that risk is higher in men, older runners, and full-marathon participants, with most events occurring in the final phase of the race or shortly after finishing. Coronary artery disease remains the predominant cause in middle-aged and older runners, whereas inherited cardiomyopathies, congenital coronary anomalies, and myocarditis are more relevant in younger athletes. We further discuss the mechanistic basis of exercise-triggered fatal events, including acute ischemia, malignant ventricular arrhythmias, myocardial fibrosis, and inflammatory myocardial injury. Current preventive approaches, including pre-participation cardiovascular screening, electrocardiography, advanced imaging, genetic evaluation in selected individuals, and rapid on-site resuscitation systems, have improved event recognition and survival outcomes, but important controversies persist regarding optimal screening intensity, interpretation of subclinical findings, and the dose-response relationship between extreme endurance exercise and cardiovascular risk. Future efforts should prioritize precision risk stratification, longitudinal evaluation of subclinical cardiovascular abnormalities, integration of wearable monitoring technologies, and standardized race-day emergency preparedness to improve the safety of long-distance running.

## 1 Introduction

Marathon running, as a high-intensity endurance activity, has a broad global participation base. Its health benefits have been widely recognized, including improved cardiovascular function and extended lifespan (1, 2). Concerns about exercise-associated sudden cardiac death (SCD), however, are not new. Pioneering studies by Maron and colleagues in the United States (3) and by Corrado, Pelliccia, and colleagues in Italy from the late 1980s onward established SCD in athletes as a distinct clinical and public-health problem and laid the foundations for modern sports cardiology (4, 5). The recent surge in public participation has further intensified attention to rare yet fatal cardiovascular events during such activities. Tragic incidents involving seemingly healthy individuals, including Canadian physicians, experiencing SCD during long-distance running have prompted profound medical reflection on the paradox that “life-prolonging activities may themselves cause death” (6). These events highlight potential cognitive gaps in assessing the risk of major adverse cardiovascular events occurring in healthy individuals during long-distance running.

Exercise-associated sudden cardiac death (SCD) has become a central issue in sports medicine and cardiovascular prevention. Despite the low absolute risk, its catastrophic nature compels us to deeply understand its epidemiological characteristics, underlying pathophysiological mechanisms, and optimize risk screening and management strategies. From a public health perspective, with the continuous growth in participation, for example, the number of marathon and half-marathon finishers in the United States from 2010 to 2023 was three times that from 2000 to 2009 (7), even an extremely low event incidence may translate into an absolute number of cases that cannot be ignored.

A key knowledge gap remains, however: existing reviews tend to conflate marathon-specific data with extrapolations from broader athletic cohorts, use loose causal language when describing the impact of high-volume endurance exercise on coronary disease, and inadequately integrate emerging marathon registries with the practical limitations of wearable monitoring. In this mini-review, we synthesize recent evidence from marathon-specific datasets, contemporary young-athlete screening cohorts, and longitudinal studies of lifelong endurance athletes to reassess the prevention of marathon-associated SCD. Specifically, we integrate the JAMA 2025 US long-distance race dataset, Japanese marathon-related cardiac arrest cohorts, and the JACC 2026 screening study by MacLachlan et al.; appraise emerging evidence from Master@Heart and MARC-2 on coronary atherosclerosis in endurance athletes; contrast US and European pre-participation screening strategies for a heterogeneous, predominantly master-aged marathon population; and evaluate the promise and limitations of wearable monitoring technologies. This synthesis aims to clarify remaining evidence gaps and provide practical considerations for safer long-distance race participation (Table 1).

**Table 1:** Representative studies on marathon-associated sudden cardiac death.

Theme	Study	Design/population (sample size, age)	Key findings	Clinical relevance
Incidence	Gerardin et al. (8)	Prospective RACE PARIS registry, >1 million runners + meta-analysis (8 registries; 16.22 million runners); mixed age, both sexes	Registry: life-threatening events 3.35/100,000; major cardiac events 2.33/100,000; cardiac arrest 1.67/100,000. Meta-analysis: cardiac arrest 0.82/100,000; mortality 0.39/100,000.	Establishes a low absolute but measurable risk during long-distance races.
Risk profile	Kim et al. (7)	US long-distance race data, 2010–2023; full + half marathons; mixed age, both sexes	Full marathon risk > half marathon; male incidence 1.12/100,000 vs. female 0.19/100,000; most events occurred late in the race or immediately after finishing; SCD incidence and case fatality decreased vs. 2000–2009.	Supports stratification by sex and event distance; demonstrates AED-era survival improvement.
Risk profile	Manabe et al. (11)	Prospective Japanese marathon study; >4 million runners; age stratified	Men accounted for the vast majority of cardiac arrest cases; incidence rose with age—0.9/100,000 (40 s), 2.6/100,000 (50 s), 5.5/100,000 (≥60).	Identifies older male runners as a priority group for pre-race assessment.
Young-athlete etiology	Petek et al. (10)	20-year retrospective NCAA cohort; multiple sports (basketball most represented); not marathon-specific; age <25 years	Predominant findings included autopsy-negative unexplained sudden death and idiopathic LV hypertrophy/HCM.	Findings inform young-athlete etiology but require caution when extrapolating to marathon runners.
Young-athlete incidence	MacLachlan et al. (5)	>100,000 young athletes screened (mean age ∼23 years); multiple sports; both sexes	SCD incidence 5.6/100,000 across multiple sports.	Demonstrates that risk in young competitive athletes is not negligible; marathon-specific data still lacking.
Pathophysiology	De Bosscher et al. (14)	Master@Heart prospective study; lifelong male endurance athletes vs. healthy non-athlete controls (low CV risk)	Lifelong endurance athletes had greater coronary plaque burden, including more proximal non-calcified plaques.	Suggests occult coronary atherosclerosis may underlie ischemic events in older runners.
Pathophysiology	Aengevaeren et al. (15)	MARC-2 longitudinal cohort; middle-aged and older athletes	Exercise intensity, rather than total volume, was associated with progression of CAC and calcified plaque burden; very high-intensity exercise correlated with greater progression.	Reinforces the unresolved dose-response debate; intensity may matter more than volume.
Screening	Vessella et al. (28)	Italian pre-participation screening programme; nearly 6,000 athletes (mostly young competitive)	Primary screening + secondary testing identified disease in ∼2.0% and SCD-risk conditions in 0.3%; cost ∼€79 per athlete.	Shows structured ECG-inclusive screening can detect occult disease with reasonable efficiency.
Exercise dose	German et al. (42)	MESA prospective multi-ethnic cohort; >5,000 community-dwelling adults	Higher physical activity was associated with greater coronary artery calcium but did not consistently translate into higher cardiovascular event rates.	Counterweight to the J-curve hypothesis; plaque morphology likely matters more than burden alone.
Emergency response	Tanaka et al. (12)	Prospective study of marathon-related SCA in Japan; rapid mobile AED system	When bystander CPR was started within 1 min and defibrillation within 3 min, 95.5% of patients achieved complete neurological recovery.	Demonstrates the critical importance of rapid AED deployment and standardized race-day systems.

AED, automated external defibrillator; CAC, coronary artery calcium; CPR, cardiopulmonary resuscitation; HCM, hypertrophic cardiomyopathy; SCA, sudden cardiac arrest; SCD, sudden cardiac death.

## 2 Methods

We searched PubMed, Embase, Web of Science, and the Cochrane Library (January 2020–March 2026), combining terms for marathon/long-distance running/endurance exercise with SCD, cardiac arrest, screening, coronary atherosclerosis, cardiomyopathy, myocarditis, CMR, and wearable devices, restricted to English and humans. Selected pre-2020 landmark studies (Maron; Corrado/Pelliccia) were included for historical context.

Two authors (X.Z. and S.L.) independently screened records, with disagreements resolved by a third author (J.P.). Eligible designs included prospective registries, cohort studies, meta-analyses, peer-reviewed consensus statements, and society guidelines; case reports, conference abstracts, animal studies, and non-cardiovascular publications were excluded. Reference lists were hand-searched.

As a mini review, we did not apply a formal risk-of-bias instrument or perform meta-analysis, but cited studies were informally weighted, giving greatest emphasis to large prospective registries, athletic cohorts, and meta-analyses, and treating cross-sectional imaging studies and expert consensus as supportive evidence. Inconsistencies between datasets were examined narratively with explicit attention to differences in event definitions, ascertainment, race-distance mix, age and sex composition, and whether populations were marathon-specific or pooled across sports.

## 3 Epidemiology and clinical characteristics of sudden cardiac death in marathon running

Marathon-associated SCD has a low absolute incidence but distinct demographic and clinical patterns. As detailed below, available studies differ substantially in case definitions, registry completeness, and athlete populations, so reported figures should be regarded as conservative, marathon-mixed approximations rather than directly comparable estimates (8–10).

A prospective registry of over one million runners in the Paris region of France demonstrated a life-threatening event rate of 3.35 per 100,000, with major cardiac events at 2.33 per 100,000 and cardiac arrest at 1.67 per 100,000 (8). A meta-analysis included in the same article, which incorporated 8 registry studies and involved 16,223,866 runners, further demonstrated that the incidence and mortality rates of long-distance race-associated cardiac arrest were 0.82 per 100,000 and 0.39 per 100,000, respectively (8).

Direct comparison of the three largest contemporary datasets, the Paris RACE registry (8), the Japanese marathon study (11), and the US 2010–2023 cohort (7), provides converging signals despite methodological heterogeneity. All three confirm male predominance, an absolute risk on the order of 0.4–1.7 per 100,000 finishers, and CAD as the dominant aetiology in master runners. They differ, however, in event definition (cardiac arrest vs. SCD), ascertainment (mandatory medical reporting, voluntary registry, or news-database hybrid), autopsy availability, and race-distance composition. Absolute incidence values are therefore not strictly interchangeable, but the qualitative concordance strengthens confidence in the identified high-risk profile (older male, full-marathon, late-race timing).

### 3.1 Master athletes (≥35 years)

Most marathon participants are aged ≥35 years. Among them, males represent a clearly identified high-risk group. A prospective Japanese observational study of over 4 million marathon participants showed that males constituted the vast majority of all cardiac arrest cases (11). US data similarly indicate that the incidence of cardiac arrest among male runners (1.12 per 100,000) is significantly higher than among females (0.19 per 100,000) (7). Age is another key factor: the incidence increases progressively with age, from 0.9 per 100,000 in runners in their 40 s, to 2.6 per 100,000 in their 50 s, and 5.5 per 100,000 in those over 60 years (11). This age-dependent trend is clearly evident in males but not statistically significant in females (11). The risk of full marathons exceeds that of half marathons (7), and most cardiac arrests occur during the final third of the race or immediately after the finish line (7, 9). Notably, the running speed of athletes who suffered cardiac arrest did not differ from the median speed of all finishers, challenging the assumption that less fit runners face higher risks (9). In this older subgroup, coronary artery disease (CAD) is the dominant cause: in the Paris registry, myocardial ischemia accounted for 11/18 confirmed cardiac arrests (8), and US 2010–2023 data also identified CAD, rather than hypertrophic cardiomyopathy, as the most common etiology (7).

### 3.2 Younger athletes (<35 years)

Etiological data for younger athletes are largely derived from broader athletic populations rather than marathon-specific cohorts. The 20-year NCAA study enrolled SCDs across many sports, with basketball most represented; consequently, its findings should be extrapolated to young marathon runners with caution (10). In that and similar young-athlete cohorts, autopsy-negative unexplained sudden death, idiopathic left ventricular hypertrophy/cardiomyopathy, and hypertrophic cardiomyopathy predominate (10). A more recent large screening study by MacLachlan et al. in over 100,000 young athletes (mean age ∼23 years) reported an SCD incidence of 5.6 per 100,000 across multiple sports (5), indicating that risk among young competitive athletes is not negligible and may, in some sport-specific contexts, even exceed that of master marathon runners. However, since this cohort spanned multiple disciplines, dedicated marathon-specific data in this age group remain scarce, and longitudinal studies focused on young long-distance runners are urgently needed.

Encouragingly, with improved event medical support, particularly rapidly deployable automated external defibrillator (AED) systems, both survival and neurologically intact survival have significantly increased. Among Japanese cases where bystander cardiopulmonary resuscitation (CPR) was initiated within 1 min and defibrillation completed within 3 min, 95.5% achieved complete neurological recovery at 1 month and 1 year (12). Compared with 2000–2009, US data from 2010 to 2023 showed lower SCD incidence (0.20 vs. 0.39 per 100,000) and lower case fatality (34% vs. 71%) (7), highlighting the importance of effective emergency action plans and immediate defibrillation.

## 4 Pathophysiological basis of marathon-associated sudden cardiac death: from coronary artery disease to myocardial pathologies

The extreme physiological stress imposed on the heart during marathon running may trigger acute events or unmask underlying cardiac conditions. The pathophysiological basis is diverse, primarily encompassing CAD, congenital coronary artery anomalies, primary cardiomyopathies, and myocarditis.

CAD constitutes the most common structural cause of SCD among middle-aged and elderly marathon runners. The relationship between long-term high-intensity endurance exercise and coronary atherosclerosis exhibits an "exercise paradox": regular exercise is the cornerstone of cardiovascular prevention (2), yet growing evidence suggests that long-term, high-volume endurance training may accelerate rather than decelerate the progression of coronary atherosclerosis (13, 14). The Master@Heart prospective study showed that lifelong male endurance athletes possessed greater coronary plaque burden, particularly more proximal non-calcified plaques, than healthy controls with similarly low cardiovascular risk (14). Longitudinal MARC-2 data further reveal that exercise intensity, rather than total volume, correlates with progression of coronary calcification: very high-intensity exercise is associated with greater advancement, while high-intensity exercise correlates with less progression (15).

Beyond classic atherosclerosis, congenital coronary artery origin anomalies represent the second leading cause of SCD among young athletes (16). Echocardiographic screening is feasible, but diagnostic criteria for minor variants such as “high take-off” remain unstandardized (17). Myocardial bridging is increasingly recognized for its potential ischemic mechanism during exercise-induced tachycardia, when shortened diastole intensifies systolic compression (18). Spontaneous coronary artery dissection (SCAD), although a recognized cause of acute coronary syndrome predominantly in younger women, has only rarely been documented as a cause of marathon-associated cardiac arrest, and its specific contribution to long-distance running events remains unquantified (19, 20). Further dedicated studies are needed to determine its true role in this population.

Among myocardial pathologies, arrhythmogenic cardiomyopathy (ACM) and myocarditis are key etiological factors, predominantly affecting athletes under 35 years. ACM is an inherited disease caused mainly by desmosomal protein gene mutations, characterized by progressive fibrofatty replacement that forms the substrate for ventricular arrhythmia (21). High-intensity exercise (as opposed to moderate physical activity) increases disease penetrance, worsens phenotypes, and may induce life-threatening ventricular arrhythmias (21). Specific genotypes such as the TMEM43 p.S358L mutation show particularly strong associations with exercise-related arrhythmic risk (22). ACM may present with a “hot phase” of chest pain and elevated cardiac enzymes, requiring differentiation from myocarditis through histology, family history, and genetic testing (23). Some authors propose that prolonged endurance exercise itself may induce ACM in predisposed individuals (i.e., exercise-induced ACM) (24).

**Figure 1:**
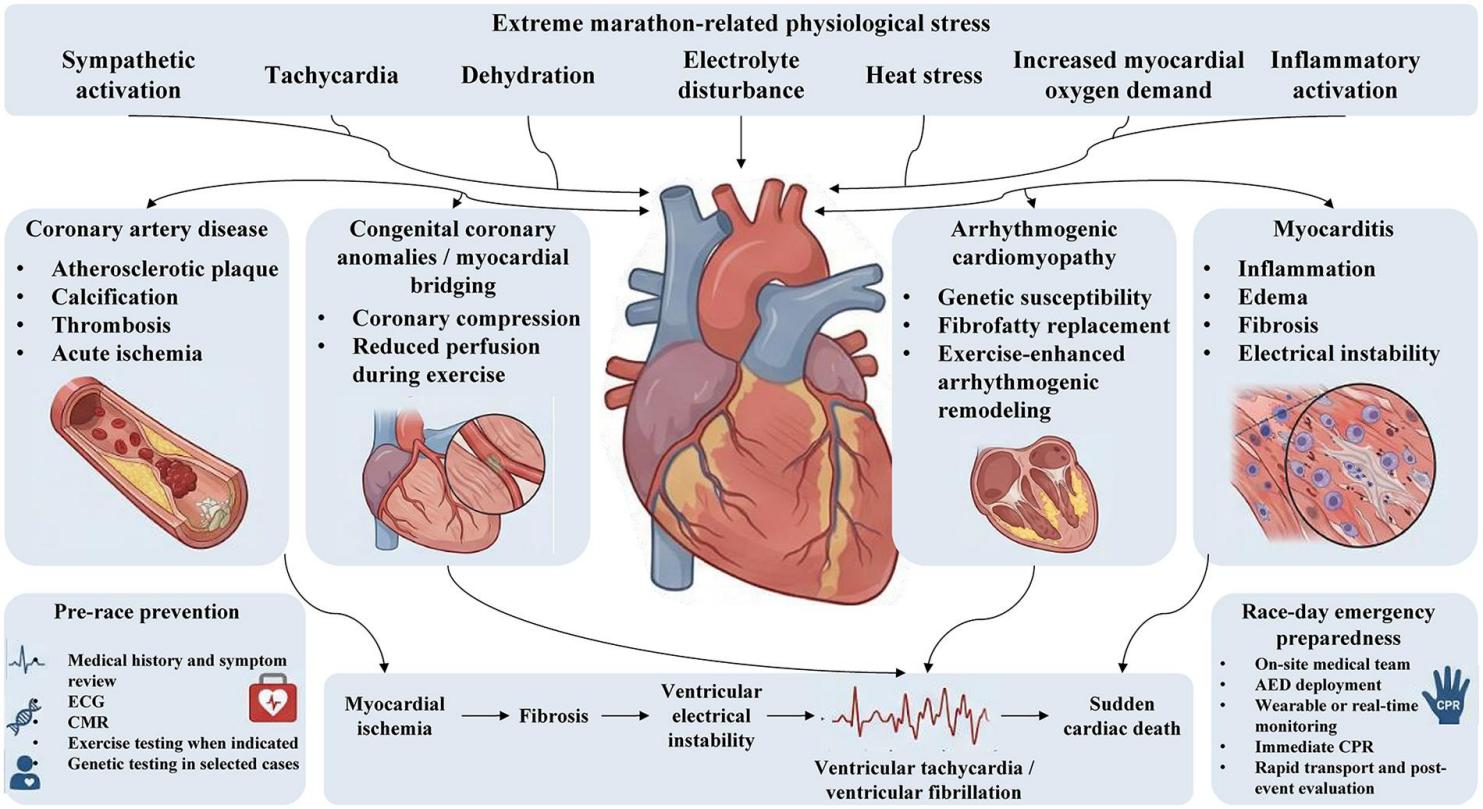
Pathophysiological mechanisms and prevention framework of marathon-associated sudden cardiac death.

Myocarditis is another significant cause of SCD in athletes (25, 26). Athletes face increased risk because of global travel-related pathogen exposure and premature training resumption during/after infection, which impairs immune function (25). The presence of myocardial inflammation, edema, and fibrosis, typically assessed by cardiac magnetic resonance (CMR), is a critical prognostic determinant (25). Distinct features characterize different stages (acute, chronic active, healing); endomyocardial biopsy retains essential diagnostic and differential value in selected cases (27) (Figure 1).

## 5 Screening, diagnosis, and stratified management strategies for cardiovascular risk in athletes

Effective prevention of marathon-associated SCD relies on systematic screening, diagnosis, and risk management. International approaches differ substantially: the European Society of Cardiology and the Italian model recommend mandatory pre-participation screening including a resting 12-lead ECG for competitive athletes, whereas the American Heart Association/American College of Cardiology recommend a 14-element history-and-physical-based evaluation, considering routine ECG screening to be of unproven cost-effectiveness in the heterogeneous US athletic population (28, 29). Importantly, mandatory cardiac screening currently exists only in selected countries (e.g., Italy, Israel) and predominantly in selected disciplines such as competitive soccer or professional cycling, and is targeted mostly at younger competitive athletes rather than the largely recreational, age-mixed marathon population (8).

### 5.1 Younger competitive athletes

The Italian preparticipation programme illustrates a structured screening pathway. In nearly 6,000 athletes, primary evaluation (history, physical examination, ECG) plus secondary testing identified disease in approximately 2.0% of seemingly healthy athletes and SCD-risk conditions in 0.3%, at approximately €79 per athlete (28). Adding exercise stress testing to the standard protocol increased diagnostic yield by 75% but raised false-positive findings (29). Premature ventricular beats detected during screening require multiparameter evaluation: site of origin, morphology, frequency, complexity, exercise response, and reproducibility all serve as critical bases for risk stratification (30, 31). The majority of premature ventricular beats in athletes are benign, but advanced workup, including CMR, cardiac CT, genetic testing, and even endocardial voltage mapping or biopsy, is warranted in high-suspicion cases (31, 32).

### 5.2 Master marathon runners

Most marathon runners are recreational adults aged ≥35 years, in whom universal mandatory ECG-inclusive screening is logistically and economically unfeasible. A pragmatic, layered approach is therefore needed: structured self-screening using validated questionnaires covering personal symptoms (exertional chest pain, syncope, dyspnea), family history of premature SCD, and conventional cardiovascular risk factors, followed by clinical evaluation only in those with positive responses or established high-risk profiles (8). For high-risk subgroups, middle-aged men, those with traditional risk factors, or symptomatic individuals, cardiac imaging may add value: CMR can identify myocardial scarring in up to 27% of asymptomatic “healthy” endurance athletes, and ischemic late gadolinium enhancement (LGE) is associated with significantly increased SCD risk (33). Non-ischemic LGE at right ventricular insertion points is tenfold more common in elite endurance athletes than in healthy controls, but its long-term clinical significance remains uncertain and may partly reflect physiological remodeling (34, 35). As an invasive technique, 3D electroanatomic mapping may identify low-voltage areas indicative of arrhythmogenic substrate, providing supplemental value when CMR is inconclusive (32, 36). Genetic testing aids diagnosis and family screening in confirmed or strongly suspected ACM or HCM, but interpretation in athletes with unclear phenotype requires caution (21, 22).

Once the disease is identified, individualized stratified management is essential. Athletes diagnosed with conditions posing SCD risk, such as ACM, severe coronary artery anomalies, or acute myocarditis, should be restricted from high-intensity competitive sport (16, 21, 25). Patients with ACM and pathogenic gene carriers should avoid high-intensity activity, but moderate exercise appears safe, and the health benefits of physical activity should not be denied (21). For runners with CAD, management requires comprehensive assessment of ischemic burden, plaque morphology, and cardiac function to design personalized training and competition plans (13).

## 6 Controversies and unanswered questions: reassessment of exercise dose, cardiac adaptation, and risk

While the benefits of regular exercise are indisputable, several controversies persist regarding the relationship between exercise dose, long-term cardiac adaptation, and cardiovascular risk.

The most central debate concerns the “J-shaped” or “inverted J-shaped” dose-response curve. Substantial evidence shows that moderate-to-vigorous activity reduces all-cause and cardiovascular mortality (1, 37, 38), whereas observational data suggest that very long-term, very high-intensity endurance exercise may have adverse cardiovascular effects (39, 40). Individuals with habitual exercise volumes ∼3 times above guideline recommendations show elevated coronary calcification progression (41). However, this finding has not consistently translated into higher cardiovascular event rates: in MESA and similar large cohorts, the calcified plaques observed in highly active individuals appear more stable and less prone to rupture than plaques of comparable volume in sedentary individuals (42). This inconsistency lies at the heart of the J-curve debate, and exercise intensity may matter more than volume: in MARC-2, very high-intensity exercise was associated with calcification progression, while high-intensity (but not extreme) exercise correlated with reduced progression (15).

Crucially, all of the evidence underpinning the “exercise paradox” is observational, and the gap between association and causation deserves explicit emphasis. Several biases plausibly distort the apparent harm of high-volume training, including healthy-survivor selection effects in cohorts of lifelong endurance athletes (14, 15). A second concern is reliance on surrogate endpoints, since most positive findings are anchored on coronary artery calcium progression rather than hard cardiovascular events, and CAC cannot distinguish stable from vulnerable plaque morphology (42). Residual confounding (diet, medications, fitness, body composition) and reverse causation (subclinical disease shaping training behaviour) cannot be excluded. To date, no randomized data and no large prospective study using hard cardiovascular endpoints have demonstrated that very high-volume endurance training causes excess events in otherwise healthy adults (40). The current evidence supports an associative signal but is insufficient to establish causation, and the public-health message that the benefits of regular exercise far outweigh its rare risks remains valid.

Differentiating the physiological “athlete's heart” from early pathological states presents substantial diagnostic challenges. Prolonged endurance training induces ventricular dilation, mild wall thickening, and functional adaptations that may overlap with early dilated or arrhythmogenic cardiomyopathy (24, 43). One study found that approximately one-sixth of elite endurance athletes demonstrated reduced ejection fraction, correlating with higher premature ventricular beat burden and genetic predisposition assessed by polygenic risk scoring (43).

Interpretation of exercise-associated cardiac biomarker elevations is also debated. Post-exercise troponin elevation has traditionally been viewed as transient and largely benign (44, 45). However, troponin T may be re-expressed in skeletal muscle disease unrelated to cardiac pathology, while troponin I is more cardiac-specific (46), and exercise-induced troponin rises may carry prognostic information in selected individuals (45). More refined algorithms are therefore needed to interpret post-exercise troponin in both ischemic and non-ischemic contexts (45).

Finally, mechanistic gaps persist: optimal management and recurrence prevention strategies for SCAD in exercise-induced events remain unclear (19, 20), as does the natural history of the ACM “hot phase” (23).

## 7 Limitations

Several limitations of this review should be acknowledged. As a narrative mini review, we did not apply a formal risk-of-bias instrument or compute pooled estimates, so selective citation bias cannot be excluded. Inclusion was restricted to English-language publications. Marathon-specific evidence remains scarce in young runners and in the long-term prognosis of CMR/CAC findings, and we therefore extrapolated cautiously from broader athletic cohorts. Heterogeneity in event definitions and ascertainment methods across cited studies limits strict quantitative comparison. Evidence on exercise-related coronary atherosclerosis, the ACM “hot phase”, SCAD, and wearable monitoring is evolving rapidly. Finally, non-cardiovascular exercise-related risks (heat stroke, exertional rhabdomyolysis, hyponatraemia) are not addressed.

## 8 Conclusion and future perspectives: building a safer long-distance running ecosystem

Marathon-associated SCD is a complex problem spanning epidemiology, pathophysiology, clinical medicine, and public health. Although absolute risk is low, occurrence correlates significantly with sex, age, underlying cardiac pathology, and exercise intensity. CAD is the principal cause in middle-aged and older runners, while inherited cardiomyopathy and myocarditis predominate in younger athletes. Modern screening, advanced imaging, electrophysiological diagnostics, and on-site emergency systems have substantially improved both detection and survival.

Future progress will depend on multiple priorities. **Research-wise**, large prospective long-term cohorts are needed to define the prognostic significance of subclinical findings such as coronary calcification and myocardial fibrosis in athletes (13, 14, 33); precise definitions of the “optimal exercise dose” for diverse populations should be established to avoid discouraging public exercise participation because of rare risks (40, 47); and gene-environment interactions in cardiac phenotype expression require investigation (43).

**Clinically**, prevention should be tailored: targeted self-screening questionnaires and selective imaging in high-risk individuals (33). Wearable devices offer continuous monitoring of heart rate and rhythm, potentially helping detect arrhythmias and abnormal haemodynamic responses (48–50). However, current wearables have important limitations: high false-positive rates in low-prevalence populations, degraded signal quality during exercise, mainly wellness rather than diagnostic regulatory approval, and no prospective evidence that wearable monitoring reduces hard cardiovascular endpoints in runners (47, 48, 50). Clinicians should therefore interpret wearable data cautiously and avoid over-reliance until robust prospective evidence becomes available.

**At the event-organization and public-health level**, standardized medical support must ensure CPR within 1 min and defibrillation within 3 min (12, 51). Runner education should improve recognition of warning symptoms (unexplained syncope, chest pain, abnormal dyspnea) and prompt timely medical evaluation (8). Clinicians prescribing exercise should also discuss the rare but real risk of fatal events as part of informed consent (6).
